# Initial assessments by psycho-oncologists: predictors of distress and support needs

**DOI:** 10.1007/s00432-025-06419-z

**Published:** 2026-01-13

**Authors:** Viktoria Ginger, Tanja Zimmermann

**Affiliations:** Department of Psychosomatics and Psychotherapy, Hannover Medical School, Carl-Neuberg-Str. 1, 30625 Hannover, Germany

**Keywords:** Initial clinical assessment, Support needs, Predictors in clinical assessment, Clinician burden

## Abstract

**Purpose:**

Beyond standardized screenings, clinical assessments by psycho-oncologists during initial consultations play a key role in guiding psychosocial cancer care. Despite their relevance, these assessments have rarely been systematically examined. The aim of this study was to analyze psycho-oncologists’ assessments of psychological distress and support needs and to identify factors influencing their assessments.

**Methods:**

In a cross-sectional study, *N* = 9 psycho-oncologists retrospectively evaluated *N* = 1048 initial psychooncological consultations. The perceived psychological distress, depression, anxiety, health literacy and support needs of patients were recorded, as well as consultation-related conditions and the psycho-oncologists’ own stress levels. Analyses involved descriptive statistics, group comparisons, correlations, and a binomial logistic regression.

**Results:**

A distress score ≥ 5 was observed by psychooncologists in 74.7% of patients; 44.5% were rated as anxious, 28.6% as depressed. Mental health diagnoses were made in 25% of cases, mainly adjustment or affective disorders. Psycho-oncological support needs were identified in 75.6% of patients. Key predictors for identifying distress and needs included patients’ desire for support (OR = 45.06), Knowledge and information about the consultation (OR = 2.66), and psycho-oncologists’ own stress levels (OR = 1.53).

**Conclusion:**

Psycho-oncological initial assessments are clinically relevant, but are subject to contextual and personal influences. The structured collection of consultation requests, information awareness, and health literacy, as well as interdisciplinary collaboration, can improve the assessment. The psychological stress of psycho-oncologists should also be systematically taken into account.

## Introduction

A cancer diagnosis represents a substantial psychological burden for those affected and is frequently accompanied by psychosocial stressors and existential concerns (Zimmermann 2023). Psycho-oncology can systematically identify and treat psychosocial stress in cancer patients (Leitlinienprogramm Onkologie Deutsche Krebsgesellschaft 2023). In order to identify patients with objectively identified needs and subjectively perceived needs, numerous standardized screening instruments (Leitlinienprogramm Onkologie Deutsche Krebsgesellschaft 2023), such as the Distress Thermometer (DT, Mehnert et al. 2006a, b, c), have been developed. These screening procedures are an integral part of certified cancer centers (Deutsche Krebsgesellschaft 2024) and form the basis for an initial assessment of patients’ psychological stress and need for support. The next priority in the further optimization of psycho-oncological identification and care is to improve care processes (Zimmermann et al. 2022). The clinical assessment of psycho-oncologists is particularly important in this context. In practice, patients who show signs of distress during screening are referred to psycho-oncology. In an initial consultation, psycho-oncologists then assess the psychological stress experienced by patients in a differentiated manner. The clinical assessment of psycho-oncologists plays a particularly important role, especially when patients do not explicitly express their stress or when stress is not clearly recognizable. This is because clinical questionnaires or structured interviews are rarely used to assess stress during initial consultations (Mehnert-Theuerkauf et al. 2023).

Instead, psycho-oncologists use their clinical expertise to assess the extent of psychological distress, explore patients’ support needs and requirements, and recommend psychosocial interventions. The expertise of psycho-oncologists goes far beyond the possibilities of psychosocial screening. The decision to provide patients with further psychotherapeutic support is heavily influenced by the assessment of psycho-oncologists (Singer et al. 2011, 2013). The initial contact is therefore not only the beginning of a therapeutic relationship, but also a decision-making point in the care process (Steven et al. 2019).

Despite this practical relevance, clinical assessments by psycho-oncologists have received little scientific attention to date. There is a lack of systematic analyses of how psycho-oncologists assess referred patients during initial contact, what criteria they use, and how these assessments influence further care recommendations. Therefore this study examines which factors influence the clinical assessment of psycho-oncologists.

Overall, previous studies on factors influencing the psychological stress of cancer patients present an inconsistent overall picture. Ernst et al. (2018) report excessive psychological stress primarily among younger cancer patients, while other authors (Köhler et al. 2015; Mehnert et al. 2018) have identified older age (> 60 years) as a factor contributing to increased stress.

There are also inconsistent findings regarding gender and the extent of stress. Several studies show that women experience higher levels of psychological stress (Pichler et al. 2019). On the other hand, Vitale et al. (2024) show that women and men are equally stressed. Women showed significantly higher depression scores, while men showed significantly higher anxiety scores. In addition, various tumor types have been associated with increased psychological stress. For example, Carlson and colleagues (Carlson et al. 2019; Mehnert and Koranyi 2018) found increased psychological stress in pancreatic and bronchial carcinomas. A meta-analysis by Batty et al. (2017) revealed significantly higher psychological distress in patients with colorectal cancer, prostate cancer, and leukemia. The stage of the disease and the treatment prognosis also paint a heterogeneous picture. Palliative and curative cancers, as well as solid and recurrent tumors have already been found to be factors influencing higher psychological distress (Roche et al. 2023; Zeissig et al. 2015). Pichler et al. (2022) found that, in addition to sociodemographic and oncological characteristics of patients (age, metastases), low patient awareness of psycho-oncology also correlates with increased psychological stress. Findings by Engler-Gross et al. (2019) also indicate that the emotional stress experienced by medical practitioners is associated with increased diagnostic assessments and deviating treatment decisions.

## Study objective

Given the ambivalence in the data, a more detailed analysis of psychological assessment and the factors influencing the assessment is important. As a professional group, psycho-oncologists are in direct, intensive contact with patients and thus have detailed insights into the potential factors influencing psychological distress. This study presents the first quantitative analysis of the initial clinical psycho-oncologists’ assessment of patients’ perceived distress and supportive care needs of patients who have been referred. The following research questions are examined:

1. How do psycho-oncologists assess the level of psychological distress (distress, depression, anxiety, mental disorders) of the referred patients?
2. What factors influence psycho-oncologists’ assessment of distress?
3. What supportive care needs do psycho-oncologists see in referred patients?

## Methods

### Study design

This is a cross-sectional, non-interventional, quantitative, written survey of psycho-oncologists regarding their initial consultations with oncology patients as a part of consultative psycho-oncological care at Hannover Medical School (MHH). The study was designed as a prospective consecutive recruitment study. Practicing psycho-oncologists were asked to evaluate initial consultations immediately after the consultation. This study was conducted as part of the multicenter main study “OptiScreen” (Zimmermann et al. 2022) and funded by German Cancer Aid. Ethical approval was obtained from the MHH ethics committee.

### Procedure

Psycho-oncologists conducted an initial consultation immediately (within 1–3 days) after patients were referred to psycho-oncology by the medical treatment team. The authors have previously published an article on referral processes (Ginger et al. 2025). The initial consultations were based on the regular consultative care provided to cancer patients in the inpatient setting and were not artificially initiated for the purposes of this study.

The initial consultations covered various departments at Hannover Medical School, which treated oncology patients on an inpatient basis. Due to institutional care structures at Hannover Medical School and a lack of cooperation, patients from the breast cancer center and pediatric oncology were not included. Patient-related data were collected and processed on the basis of prior general consent, in accordance with data protection regulations. No additional, specific consent was obtained.

The study included initial inpatient consultations between March 2020 and December 2022. No information was available on undocumented initial consultations. The inclusion criteria were (1) the presence of an initial/suspected or recurrent oncological disease at the time of the initial consultation and (2) sufficient cognitive capacity and a minimum level of linguistic ability to participate in the initial consultation.Repeat or follow-up psycho-oncological consultations were excluded. Initial consultations with a duration of less than five minutes were excluded due to insufficient informational content.

### Measurement

Using the specifically developed “consultation-assessment-questionnaire”, psycho-oncologists were instructed to document the process of the initial consultation immediately after the session, as well as the patients’ sociodemographic and oncological characteristics. Sociodemographic and oncological data that could not be explored during the consultation were obtained from the medical records. The primary focus of the “consultation-assessment-questionnaire” was the assessment of clinical distress (distress, depression, anxiety, mental disorder) and the supportive care needs of referred patients. In addition, the perceived openness of patients, their level of information, their health literacy, and their request for consultation were evaluated. Psycho-oncologists were also asked to report their own psychological distress during the consultation. The items and response scales of the questionnaire are presented in Table 1.

#### Measurement of mental stress

Immediately after the initial consultation, psycho-oncologists assessed the psychological stress of patients using the NCCN Distress Thermometer (DT, Mehnert et al. 2006a, b, c). The DT is a validated single-item screening that measures the subjective level of psychological distress on an analog scale from 0 “no distress” to 10 “extreme distress.” A score of ≥ 5 indicates clinically relevant distress and indicates a need for supportive care. For the external assessment, a linguistic adjustment was made, see Table 1.

To assess patients’ depressive and anxiety symptoms, psycho-oncologists completed the Patient Health Questionnaire–2 (PHQ-2, Löwe 2005) and the Generalized Anxiety Disorder–2 scale (GAD-2, Kroenke et al. 2007). The PHQ-2 assesses core symptoms of depression, whereas the GAD-2 captures symptoms of anxiety. Cut-off scores of ≥ 3 (response scale ranging from 0 to 3) indicate clinically relevant depressive or anxiety symptoms. The wording of the items was adapted for external assessment.

Patients’ fear of progression was assessed using items 1, 9, 11, and 12 of the short form of the Fear of Progression Questionnaire (German PA-F-KF 12; FoP-Q-SF; Mehnert et al. 2006a, b, c). The FoP-Q-SF assesses fear of disease progression across several domains (e.g., partnership/family, work, loss of autonomy) using a 5-point Likert scale ranging from 1 to 5. Higher scores indicate greater fear of progression.

#### Measurement of mental health support

The perceived supportive care needs were assessed with reference to the Supportive Care Needs Survey (SCNS, Boyes et al. 2008; Lee et al. 2022). Psycho-oncologists were asked to indicate the patient’s primary area of supportive need (psychological, physical, informational, or social). In addition, psycho-oncologists valuated whether further psycho-oncological support was required and, if so, the specific type of supportive care needs (see Table 1).

**Table 1:** “consultation-assessment-questionnaire”. Contents, items, and response scales

Contents	Items	Response scales
*Sociodemographic data*		
Gender	Female/male	Single selection
Age		Open question
*Oncological data*		
Tumor types		Open question
Type of diagnosis	Initial diagnosis/secondary diagnosis: recurrence, additional oncological diagnoses	Single selection
Treatment prognosis	curative/non-curative: palliative, supportive	Single selection
*Distress-Screening prior to referral to psycho-oncology*		
Screening of Distress (DT ^a^)	“Screening present?”	Single selection
DT-Score		Open question
Patients request for consultation	“Is the consultation requested by the patient?”	Single selection
*Level of information*		
Receipt of information about the consultation	“Patient informed about the consultation in advance?”	Single selection
*Openness*		
Patient’s openness toward psycho-oncological support	“How would you assess the patient’s openness toward psycho-oncological support?”	1 “very low” to 5 “very high”
*Consultation process*		
Duration of the consultation		Open question
Consultation partner	Patient alone/patient and related persons	Single selection
Assessment of contextual factors	“How do you evaluate the contextual factors (organization, materials, facilities, patient availability, documentation, etc.) of the consultation?”	1 “inadequate” to 5 “very high quality”
*Multiprofessional exchange*		
Quantity	“Was there an exchange with the treatment team regarding this patient?”	Single selection
Quality	“How do you evaluate the exchange with the treatment team?”	1 “inadequate” to 5 “very high quality”
*Clinical assessment by psycho-oncologists*		
*Distress*		
DT^a^	“Please circle the number that best describes how you perceive the patient’s symptoms.”	0 “no stress” to 10 “extremely stressed”
*Depression*		
PHQ-2 ^b^	“In your opinion, how severely was the patient restricted over the last 2 weeks?”	0 “not at all” to 3 “nearly every day”
	Item 1: Little interest or pleasure in doing things	
	Item 2: Feeling down, depressed or hopeless	
*Anxiety/fear of progression*		
GAD-2 ^c^	“In your opinion, how severely was the patient restricted over the last 2 weeks?”	0 “not at all” to 3 “nearly every day”
	Item 1: Feeling nervous, anxious or on edge	
	Item 2: Not being able to stop or control worrying	
PA-F-KF ^d^	“How high do you estimate the patient’s concerns about the future regarding his illness?”	0 “never” to 4 “very often”
	Item 1: Anxiety about the further course of the disease	
	Item 9: Fear of drastic medical measures	
	Item 11: Fear of what will happen to his family if something happens to him	
	Item 12: Fear of missing work due to illness/no longer being as productive	
*Current mental disorder*		
ICD-10-GM ^e^	“Is there a current F diagnosis?” (specify including diagnostic certainty)	Open question
*Health literacy*		
HLS-EU-Q16 ^f^	“In your opinion, how easy is it for the patient…”	0 “very difficult” to 3 “very easy”
	Item 2: “…find out where he can get professional help when he is ill?”	
	Item 8: “…information about support options for mental health issues, such as depression?”	
Health literacy in own need for psycho-oncological support	“How would you rate the patient’s ability to recognize their own need for psycho-oncological/psychotherapeutic support?”	1 “very low” to 5 “very high”
*Supportive care needs*		
SCNS ^g^	Please indicate where you see the patient’s greatest need for support:”	Multiple selection
	Mental health issues	
	Communication with the treatment team	
	Physical complaints (e.g., pain)	
	Partnership/sexuality/family life	
	Access to other counseling services (e.g., pension benefits)	
	Other	
Assessment for supportive care needs	“In your opinion, is further psycho-oncological/psychotherapeutic support currently advisable for the patient?”	Single selection
Type of support	Follow-up consultations	Multiple selection
	Psychotropic drugs	
	Psycho-oncological consultations during outpatient oncological treatment	
	Psycho-oncological consultations during rehabilitation	
	Outpatient psychotherapy	
	Inpatient psychotherapy	
	Couples therapy/couples counseling	
	Other	
*Distress of psycho-oncologists*		
Distress (DT ^a^) of psycho-oncologists during the consultation	“Please circle the number that best describes how much distress you have been.”	0 “no stress” to 10 “extremely stressed”

^a^Mehnert et al. (2006a, b, c). ^b^ Löwe (2005). ^c^ Kroenke et al. (2007). ^d^ Mehnert et al. (2006a, b, c). ^e^ ICD-10-GM

(German Modification Chapter V F diagnoses; Dilling and Freyberger 2016). ^f^ Jordan and Hoebel (2015). ^g^ Boyes et al. (2008); Lee et al. (2022)

### Statistical analysis

The analyses were conducted using IBM SPSS Statistics 28. Descriptive statistics (frequency distributions, prevalences, means, and percentages) were calculated for the total sample as well as for gender- and age-specific analyses. Group differences (gender, age, tumor entity, psychological distress) were examined using t-tests, chi-square tests, and ANOVA. Age-specific stratification followed Meeker et al. (2017). Associations were assessed using Pearson correlations. Binary logistic regression was used to identify influencing factors; significant predictors were reported as odds ratios (OR). Effect sizes were based on Cohen (1988). The level of significance was set at *p* = .01 to minimize the risk of false-positive results.

## Results

### Sample characteristics

#### Psycho-oncologists

All psycho-oncologists (*N* = 9) were female and had a mean age of 30.4 years (SD = 6.7, range = 22–42). 66.6% (*n* = 6) of the psycho-oncologists worked > 15 h per week in the psycho-oncological consultation service. The average duration of employment of psycho-oncologists was 28.6 months (SD = 28.1, range = 1–89). All (*N* = 9) had a degree in psychology. 88.9% (*n* = 8) were licensed psychotherapists or were undergoing further training. 66.6% (*n* = 6) had additional training in psycho-oncology or were undergoing such training.

88.9% (*n* = 8) of the psycho-oncologists participated regularly in supervision. 62.5% (*n* = 5) engaged in regular continuing education (at least biweekly). The psychological distress (DT) of psycho-oncologists during the initial consultation was low, with a mean of M = 1.53 (SD = 1.47, range: 0–10).

#### Patients

From an annual population of approximately 2000 newly diagnosed oncology patients at MHH, *N* = 1048 initial patient consultations were documented during the study period (see Table 2, Notes). No information is available on patients who declined the initial consultation when contacted by psycho-oncologists.

Of the *N* = 1048 patients, 51.1% (*n* = 536) were female and 46.7% (*n* = 489) were male; gender information was missing for *n* = 23 patients. The mean patient age was 60.5 years (SD = 14.5 years). The most common tumor types were gastrointestinal tract tumors, accounting for 28.1% (*n* = 289). All sociodemographic and oncological characteristics are presented in Table 2.

**Table 2:** Sociodemographic, oncological, and distress-screening data for the total sample and by gender

Age^a^ (range: 18–93)		Total*	Female	Male
			M (SD), n	
18–93 years		60.57 (14.53), 1031	60.24 (14.04), 532	61.00 (15.15), 484
18–39 years		31.60 (5.95), 109	31.95 (5.85), 56	31.10 (6.07), 52
40–65 years		55.41 (6.48), 482	55.67 (6.23), 258	55.08 (6.72), 213
65 + years		71.12 (4.26), 357	70.96 (4.26), 181	71.27 (4.28), 173
80 + years		83.14 (2.893), 83	82.51 (2.25), 37	83.65 (3.25), 46

Tumor types	Classification^b^		% (n)	
Head and neck	C00-C14	4.8 (49)	3.3 (17)	6.7 (32)
Gastrointestinal tract	C15-C26	28.1 (289)	26.2 (137)	29.5 (142)
Thorax	C30-C39	9.3 (95)	10.1 (53)	8.3 (40)
Skin	C43-C44	6.3 (65)	7.8 (41)	4.6 (22)
Soft tissue/mesothelium	C45-C49	5.2 (53)	5.0 (26)	5.4 (26)
Urogenital tract	C64-C68	4.6 (47)	3.6 (19)	5.6 (27)
Hematological	C81-C96	15.3 (157)	13.8 (72)	16.8 (81)
Other tumors (*n* < 4.5%)		17.8 (182)	18.0 (94)	18.1 (87)
Others, non-oncological diagnoses		8.8 ( 90)	12.2 (64)	5.0 (24)

Type of diagnosis				
Initial diagnosis		66.6 (679)	64.0 (332)	69.7 (333)
Secondary diagnosis: recurrence		21.6 (220)	22.0 (114)	20.7 (99)
Secondary diagnosis: additional oncological diagnoses		11.8 (120)	14.1 (73)	9.6 (46)

Treatment prognosis^c^				
Known		68.5(708)	70.0 (369)	66.3 (321)
Curative		39.6 (409)	43.3 (228)	36.0 (174)
Non-curative: palliative, supportive		60.4 (625)	56.7 (299)	64.0 (310)
Unknown		31.5 (326)	30.0 (158)	33.7 (163)

Distress-Screening (DT^d^) prior to referral to psycho-oncology				
Distress Screening (DT)		27.5 (286)	29.6 (158)	24.6 (119)
Patients‘ request for consultation		69.2 (724)	73.0 (390)	65.6 (321)

			M (SD), n	
DT-Score		6.09 (2.44) 249	6.12 (2.37) 137	6.06 (2.52) 107

*Notes*: ^a^ Age stratification according to Meeker et al. (2017). ^b^ Classification of tumor types according to ICD-10-GM, Chapter II C diagnoses (Dilling and Freyberger 2016). ^c^ Classification of treatment prognoses according to the German Cancer Society (Deutsche Krebsgesellschaft and Deutsche Krebshilfe 2018). ^d^ Distress Thermometer (Mehnert et al. 2006a, b, c)

* Overall population: primary incident cancer cases at the Oncology Center of Hannover Medical School according to the counting methodology of the German Cancer Society (Deutsche Krebsgesellschaft and Deutsche Krebshilfe 2018): Year 2020–2022 (2020: *n* = 2057; 2021: *n* = 2023; 2022: *n* = 1842)

### Psychological distress assessment by psycho-oncologists

#### Distress

Patient distress (DT) was rated by psycho-oncologists with a mean of M = 5.68 (SD = 1.92, range: 0–10, *n* = 1040). 74.7% (*n* = 777) scored above the cut-off value of ≥ 5. 25.3% (*n* = 263) were rated as “not distressed” or “slightly distressed”. All descriptive statistics and group comparisons are presented in Table 3.

Women (M = 5.87, SD = 1.88, *n* = 533) were perceived as more psychologically distressed than men (M = 5.51, SD = 1.95, *n* = 485), *t* (1016) = 3.051, *p* < .002, d = 0.191. A significant effect of age on perceived distress was observed, F(3) = 3.780, *p* = .010, ηp^2^ = 0.011, *n* = 1025, f = 0.10. Post hoc tests with Bonferroni correction showed the lowest psychological distress among patients aged 80 years and older.

**Fig. 1:**
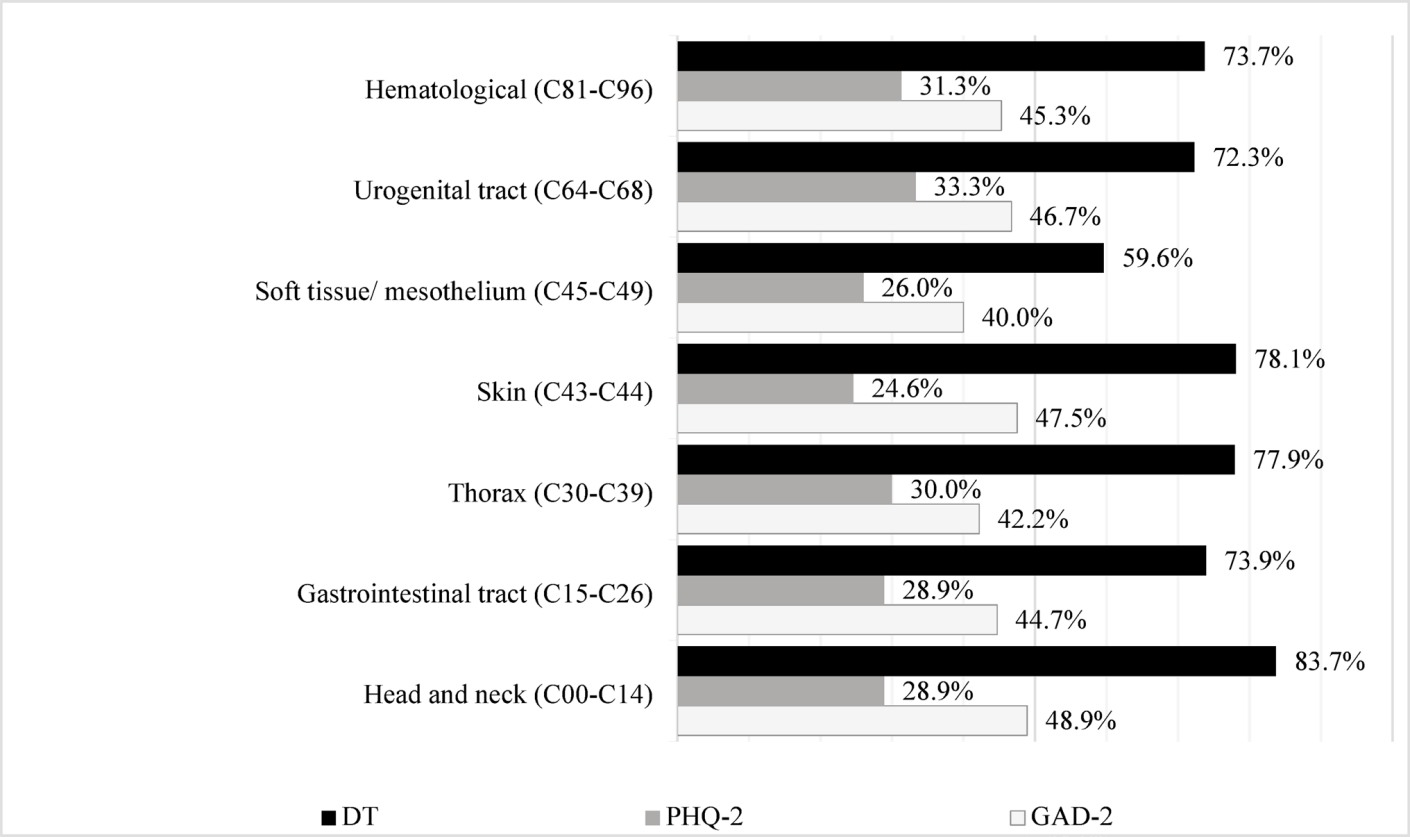
Psychological distress (%) in the most common tumor types. Assessment by psycho-oncologists. *Notes*: Psychological distress above Cut-Off Score: DT ≥ 5, PHQ-2 ≥ 3, GAD-2 ≥ 3. Classification of tumor types according to ICD-10-GM, Chapter II C diagnoses (Dilling and Freyberger 2016)

In comparisons across tumor types, psycho-oncologists most frequently reported distress and anxiety in patients with head and neck cancers (distress 83.7%, *n* = 41; anxiety: 48.9%, *n* = 22); see Fig. 1.

Group differences were observed between patients with a curative (M = 5.48, SD = 1.91, *n* = 407) and a non-curative (M = 5.80, SD = 1.91, *n* = 620) treatment prognosis. According to psycho-oncologists’ assessments, patients with non-curative treatment prognoses showed higher levels of distress, *t* (1025) = − 2.631, *p* = .009, d = 0.168. 93.0% (*n* = 596) of patients who requested a consultation were rated as distressed (DT ≥ 5). 7.0% (*n* = 45) were rated as not distressed despite requesting a consultation. In comparison between patients who requested a consultation (M = 6.36, SD = 1.43, *n* = 641) and patients without a consultation request (M = 3.95, SD = 1.93, *n* = 286), significant differences were observed. From the psycho-oncologists’ perspective, patients who requested a consultation showed higher levels of distress, *t* (430) = − 18.857, *p* < .001, d = 1.50.

The distress assessment by psycho-oncologists (M = 5.68, SD = 1.92, *n* = 1,040) correlated with patients’ self-assessment distress (M = 6.06, SD = 2.44, *n* = 249), *r* = .718, *p* < .001, *n* = 249. Psycho-oncologists’ assessment of depression (PHQ-2: *r* = .392, *p* < .001, *n* = 236) and anxiety (GAD-2: *r* = .393, *p* < .001, *n* = 236) also correlated with patients’ self-assessment distress.

#### Depression

Overall, 28.6% (*n* = 284) of patients were rated by psycho-oncologists as having elevated depressive symptoms (PHQ-2). In a group comparison between patients with a curative (M = 1.86, SD = 1.28, *n* = 398) and a non-curative (M = 2.25, SD = 1.40, *n* = 581) treatment prognosis, patients with non-curative prognoses were perceived as more depressed, PHQ-2: *t* (977) = − 4.456, *p* < .001, d = 0.28. Psycho-oncologists most frequently recorded elevated depression scores in patients with urogenital tract tumors (33.3%, *n* = 15); see Fig. 1. All PHQ-2 values and group comparisons are presented in Table 3.

#### Anxiety

44.5% (*n* = 441) of patients were rated by psycho-oncologists as experiencing significant anxiety. Women (M = 2.66, SD = 1.54, *n* = 511) were perceived as more anxious than men (M = 2.39, SD = 1.45, *n* = 465), GAD-2: *t* (974) = 2.763, *p* = .006, d = 0.177. Patients with non-curative treatment prognoses (M = 2.64, SD = 1.51, *n* = 579) showed higher anxiety levels than those with curative prognoses (M = 2.32, SD = 1.47, *n* = 398), GAD-2: *t* (975) = − 3.231, *p* < .001, d = 0.21. Psycho-oncologists rated “anxiety regarding the further course of the disease” (47.9%, *n* = 397) and “fear of what will happen to one’s family if something happens to the patient” (33.1%, *n* = 273) as particularly pronounced (PA-F-KF). According to psycho-oncologists’ assessments, patients aged 80 years and older (M = 10.93, SD = 2.95, *n* = 68) showed the lowest level of progression anxiety, F (3) = 7.832, *p* < .001, ηp² = 0.028, *n* = 820. The highest progression anxiety was observed among patients aged 40–65 years (M = 12.85, SD = 2.94, *n* = 416).

**Table 3:** Descriptive statistics and group comparisons of distress, depression, and anxiety by gender, age, and oncological data. Assessment by psycho-oncologists

Total	Gender		Age in years				Type of diagnosis ^a^		Treatment prognosis ^b^	
	Female	Male	18–39	40–65	65+	80+	Initial	Second	Curative	Non-curative
M (SD) n										
*DT (range: 0–10)*										
5.68 (1.92) 1040	5.87 (1.88) 533	5.51 (1.95) 485	5.69 (1.81) 108	5.83 (1.89) 511	5.65 (2.00) 323	5.07 (1.93) 83	5.98 (1.97) 676	5.69 (1.82) 336	5.48 (1.91) 407	5.80 (1.91) 620
	p*									
	0.002		0.010				0.911		0.009	
*PHQ-2 (range: 0–6)*										
2.10 (1.37) 1040	2.16 (1.36) 512	2.04 (1.38) 466	1.94 (1.16) 104	2.12 (1.41) 490	2.21 (1.44) 309	1.86 (1.05) 76	2.05 (1.37) 651	2.21 (1.34) 317	1.86 (1.28) 398	2.25 (1.40) 581
	p*									
	*0.181*		*0.115*				*0.078*		*0.001*	
*GAD (range: 0–6)*										
2.52 (1.50) 990	2.66 (1.54) 511	2.39 (1.45) 465	2.47 (1.37) 104	2.61 (1.48) 490	2.51 (1.60) 307	2.20 (1.29) 76	2.50 (1.52) 651	2.57 (1.46) 315	2.23 (1.47) 398	2.64 (1.51) 579
	p*									
	*0.006*		*0.142*				*0.534*		*0.001*	

* Independent-samples t-tests were used to examine group differences by gender, type of diagnosis, and treatment prognoses. One-way analysis of variance (ANOVA) was used to examine group differences by age. ^**a**^ Classification of diagnosis types into oncological categories: “initial diagnosis“ and “secondary diagnosis“ (recurrence, additional oncological diagnoses). ^**b**^ Classification of treatment prognoses into “curative” and “non-curative” (palliative, supportive)

#### Mental disorders

In 25.0% (*n* = 257) of referred patients psycho-oncologists documented a current mental disorder (ICD-10-GM, Chapter V F diagnoses; Dilling and Freyberger 2016). 49.6% (*n* = 119) of the diagnoses were classified as suspected, and 41.7% (*n* = 100) as confirmed diagnoses. The most frequent diagnoses were affective disorders (28.2%, *n* = 71), adjustment disorders (40.1%, *n* = 101), and anxiety disorders (8.3%, *n* = 21).

In 15.2% (*n* = 156) of cases, a mental disorder had already been present prior to the cancer diagnosis. The most common pre-existing disorders were affective disorders (47.3%, *n* = 62), anxiety disorders (18.3%, *n* = 24), and substance use disorders (9.2%, *n* = 12).

No differences were found between women and men, χ² (1) = 0.720, *p* = .396, *n* = 773. No differences were observed between age groups, χ² (3) = 2.378, *p* = .498, *n* = 774. There were no differences between patients with an initial diagnosis and those with a secondary diagnosis, χ² (1) = 2.793, *p* = .095, *n* = 770, nor between patients with curative and non-curative prognoses, χ² (1) = 4.250, *p* = .039, *n* = 779.

Patients who were aware of their treatment prognosis had a current mental disorder in 29.3% (*n* = 159) of cases. In contrast, 39.2% (*n* = 93) of patients who were not aware of their treatment prognosis had a current mental disorder, χ² (1) = 7.392, *p* = .007, *n* = 779.

### Factors influencing the assessment of psycho-oncologists

A binary logistic regression was used to examine predictors influencing psycho-oncologists’ assessments (see Table 4). The analysis included sociodemographic and oncological patient data, information on the consultation process (duration of the consultation, participants, exchange with the multiprofessional team, contextual factors: e.g. noise), information on prior distress screening, and the need for a psycho-oncological consultation (request for a consultation, prior information about the consultation taking place). In addition, psycho-oncologists’ assessments of patients’ health literacy, patients’ openness toward psycho-oncology, and the psycho-oncologists’ own psychological distress were included in the analysis.

The regression model was significant, χ² (16) = 144.78, *p* < .001, *n* = 1048. The explained variance was very high (R² = 0.720). The Hosmer–Lemeshow test indicated good model fit, χ² (8) = 9.82, *p* = .278. The overall percentage of correctly classified cases was 91.5% (sensitivity: 96.5%; specificity: 77.1%).

Patients’ health literacy (*p* < .001) and psycho-oncologists’ own psychological distress (*p* = .006) were significant predictors of distress ratings. Lower patient health literacy was associated with a 70% lower likelihood of being rated as clinically distressed, OR = 0.30 (95% CI [0.20–0.43]). An increase in psycho-oncologists’ own psychological distress increased the likelihood of patients being perceived as clinically distressed by 53%, OR = 1.53 (95% CI [1.13–2.06]). The variable “patients’ receipt of information about the consultation” increased the likelihood of the rating by a factor of 2.66 (*p* = .004). The variable “patients’ request for a consultation” increased the likelihood of the rating by a factor of 45.06 (*p* < .001).

**Table 4:** Predictors influencing psycho-oncologists’ distress assessments for total sample (*N* = 1048)

Predictor	Classification	*p*	OR	95% CI
*Sociodemographic data*				
Gender		0.847	–	0.50–1.76
Age		0.590	–	0.97–1.01
*Oncological data*				
Tumor types	Initial diagnosis/secondary diagnosis	0.059	2.02	0.97–4.23
Type of diagnosis	Known/unkonwn	0.170	–	0.76 − 4.50
Treatment prognoses	Curative/non-curative	0.186	–	0.22–1.33
*Current mental disorder* ^a^				
ICD-10-GM Chapter V F diagnoses	Diagnosed/not diagnosed	0.067	2.21	0.94–5.19
*Distress-Screening prior to referral to psycho-oncology* ^b^				
Screening of Distress (DT)	Present/not present	0.465	–	0.39–1.52
Patients‘ request for consultation	Requested/declined	> 0.001 *	45.06	22.03–92.18
*Level of information* ^a^				
Receipt of information about the consultation	Informed/not informed	0.004 **	2.66	1.36–5.22
*Openness* ^a^				
Patient’s openness toward psycho-oncological support		0.089	–	0.95–1.99
*Health literacy* ^a^				
Health literacy in own need for psycho-oncological support		< 0.001 *	0.30	0.20 − 0.43
*Consultation process* ^a^				
Duration of the consultation		0.537	–	0.98–1.02
Consultation partner	Patient alone/patient and related persons	0.280	–	0.48–11.94
Assessment of contextual factors (organization, materials, facilities, patient availability, documentation, etc.)		0.431	–	0.67–1.18
*Multiprofessional exchange* ^a^				
Quantity	Conducted/not conducted	0.962	–	0.46–2.05
*Distress of psycho-oncologists* ^a^				
Distress (DT) of psycho-oncologists during the consultation		0.006**	1.53	1.13–2.06

^a^Assessment by psycho-oncologists. ^b^ Assessment by patients

* *p* = significant. ** *p* = trend-level significance

### Assessment of supportive care needs by psycho-oncologists

**Fig. 2:**
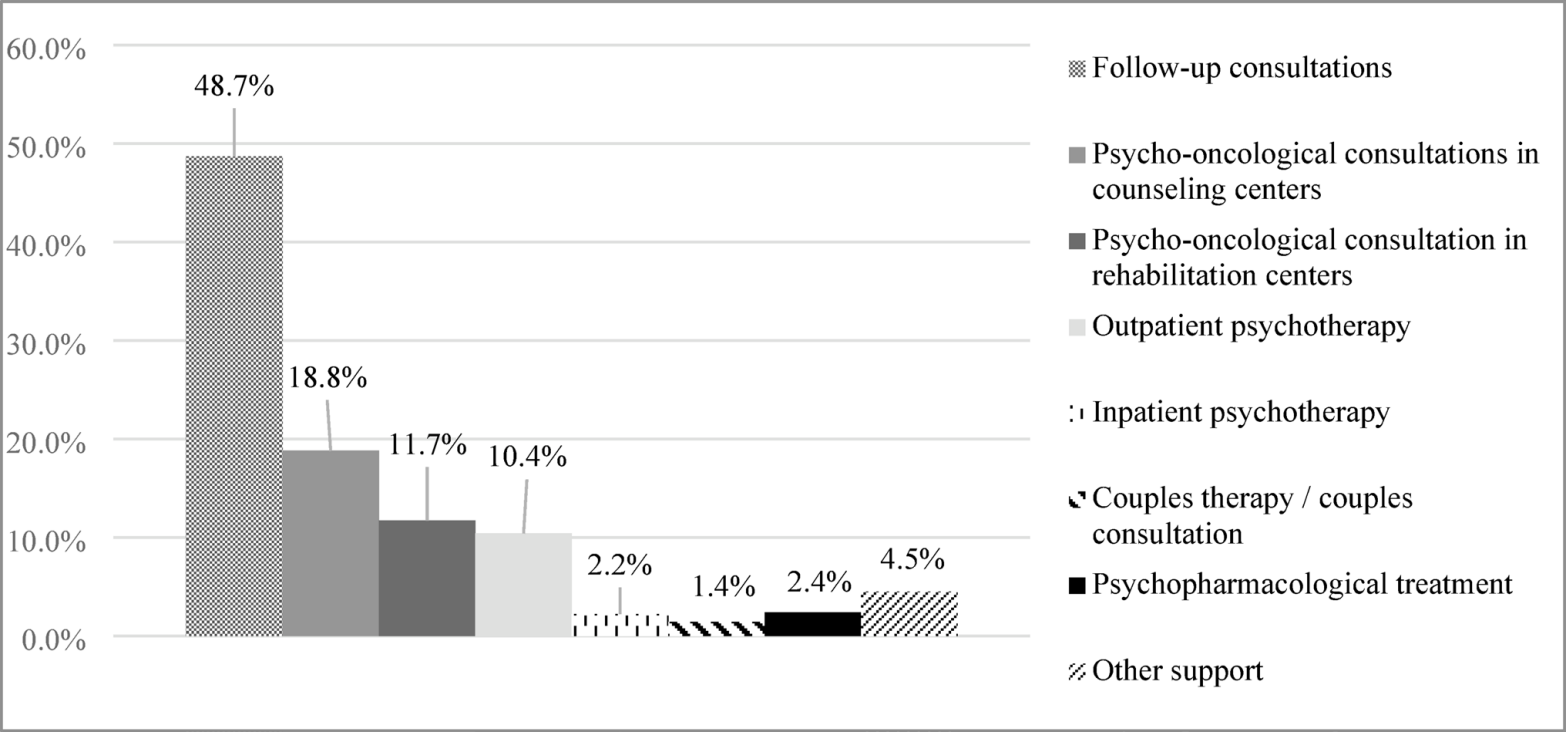
Recommendations for supportive care needs (%) for the total sample (*n* = 756). Assessment by psycho-oncologists. *Notes*: Multiple response set

The perceived distress and the recommended level of support were significantly associated: χ² (1) = 125.631, *p* < .001, *n* = 1012. For 75.6% (*n* = 743) of all referred patients (*N* = 1048), psycho-oncologists recommended further psycho-oncological or psychotherapeutic support. Psycho-oncologists reported having scheduled follow-up consultations with 51.6% of patients (*n* = 537). The most frequently recommended psycho-oncological interventions were follow-up psycho-oncological consultations (77.8%, *n* = 588) and consultations at oncological counseling centers (30.1%, *n* = 227); see Fig. 2. No gender differences were found regarding the scheduling of follow-up consultations, χ² (2) = 2.620, *p* = .106.

Differences were found between age groups and the recommended level of support, χ² (6) = 16.512, *p* = .011, V = 0.127. A Post hoc analysis showed a near-significant difference in the group aged 40–45 years. In 75.0% (*n* = 380) of patients aged 40–65 years, further psycho-oncological support was recommended, *p* = .059, *n* = 507.

Patients aged 80 years and older were recommended significantly less support (61.4%, *n* = 51), *p* = .002, *n* = 83.

Patients with non-curative prognoses were significantly more advised to receive further psycho-oncological support than curative patients, χ² (1) = 20.265, *p* < .001, *n* = 972, V = 0.144. According to psycho-oncologists, “psychological complaints” (35.6%, *n* = 615), even more than “physical complaints” (33.0%, *n* = 570), represented the greatest need for support. No differences were found between genders with regard to support domains (SCNS): χ² (6) = 0.816, *p* = .366, *n* = 900. Only “social consultations” were recommended more often to men (25.5%, *n* = 108) than to women (18.0%, *n* = 86): χ² (6) = 14.07, *p* = .029, *n* = 900, V = 0.091.

Psycho-oncologists most frequently identified support for “psychological problems” (67.4%, *n* = 615) and “support for physical problems” (e.g., assistance with pain management; 62.4%, *n* = 570) as the largest areas of psychosocial support needs among patients (SCNS).

## Discussion

74% of the patients were assessed by psycho-oncologists as experiencing distress above the clinical threshold. 44% of all patients exhibited clinically relevant anxiety symptoms, and 28% showed clinical depressive symptoms. In 25% of cases, a current mental health diagnosis was assigned, most commonly adjustment disorders or affective disorders. These findings are consistent with national prevalence data indicating that approximately one third of cancer patients experience mental disorders (Goerling et al. 2024; Leitlinienprogramm Onkologie Deutsche Krebsgesellschaft 2023).

The agreement between patients’ self-assessments and psycho-oncologists’ assessments supports the validity of psycho-oncological evaluations and thus makes a substantial contribution to the identification of distressed patients.

Overall, from the perspective of psycho-oncologists, only a limited number of sociodemographic and oncological patient characteristics were associated with higher psychological distress. Women were assessed as more anxious than men, no gender-specific differences were observed with regard to depressive symptoms or psychiatric diagnoses, consistent with similar findings reported by Mehnert et al. (2018). In terms of age, psycho-oncologists perceived patients aged 40–65 years as the most highly distressed, in line with Peters et al. (2020). One possible explanation is that individuals in this age group may experience destabilization across multiple central domains of life and are therefore perceived by psycho-oncologists as particularly burdened.Previous studies confirm that threats to employability, work-related functional impairments (Duijts 2017) and high family responsibilities (Leitlinienprogramm Onkologie Deutsche Krebsgesellschaft 2023) constitute distress for patients. In the present study, the lowest levels of distress were observed among patients over 80 years of age, who were also less frequently recommended supportive interventions.

However, the cross-sectional design of this study does not allow conclusions to be drawn about trajectories of distress within age groups. In addition, detailed information on life-contextual factors, such as employment status or family responsibilities, is lacking, although these factors may contribute to the presumed vulnerability of certain age cohorts. Future studies should therefore employ longitudinal designs to capture social-structural and biographical variables in order to better understand age-related differences.

In comparisons across tumor types, psycho-oncologists assessed patients with oral cavity tumors as well as those with gastrointestinal tract tumors as highly distressed. It may be assumed that oral cavity tumors are associated with simultaneous aesthetic, communicative, and functional impairments, which are particularly apparent to psycho-oncologists during the initial consultation. This is consistent with findings reported by Nayak et al. (2022) and Jimenez-Labaig et al. (2024).

For bladder and prostate carcinomas, studies likewise report high prevalence of anxiety, depressive symptoms, and persistent distress (Bahlburg et al. 2024). Possible contributing factors include stigma and functional impairments such as incontinence, sexual dysfunction, and loss of intimacy, which psycho-oncologists associate with high levels of distress.

In comparison, patients with non-curative treatment prognoses were assessed as more highly distressed than patients receiving curative treatment. One possible explanation is that, psycho-oncologists perceive more complex dimensions of distress, such as persistent losses of autonomy, changes in family roles (van Roij et al. 2019), and existential concerns (Philipp et al. 2025) in consultations with non-curative patients.

Nevertheless, the referral rate was higher among patients at initial diagnosis (66%) compared with patients with recurrent disease (33%). To investigate the reasons for referrals among specific patient groups, future studies should place greater emphasis on institutional differences, cooperation structures, and the sensitivities of individual oncological specialties. This research group has previously pointed out that, in the absence of personnel training and structured tools (e.g., distress screening), assessments of distress and referrals made by the medical team tend to be imprecise or unsystematic (Dreismann et al. 2021; Faller and Schmidt 2004; Keller et al. 2004; Senf et al. 2019) .

Overall, this study demonstrates that, from the perspective of treating psycho-oncologists, cannot be explained by gender, age, or tumor type, but is instead largely transdiagnostic (Bultz and Johansen 2011; Mehnert et al. 2014). Instead, the predictors “receipt of information about the consultation” (OR = 2.66) and “patients request for consultation” (OR = 45.06) significantly influenced the likelihood of being assessed as highly distressed. These findings support previous evidence (Hack et al. 2005) showing that actively seeking psychosocial support is associated with a higher probability of receiving care and with improved treatment adherence.

Likewise low health literacy was associated with a significantly increased risk of being classified as clinically distressed. These findings are consistent with the results of McCaffery et al. (2013), who demonstrated an association between low health literacy, increased distress, and reduced coping strategies. In the context of the present study, perceived patient health literacy and participation appear to play a particularly central role in how patients’ levels of distress are assessed by psycho-oncologists. Consequently, these factors also substantially influence providers’ support recommendations.

Another important finding of this study is that with increasing levels of personal distress among psycho-oncologists, the likelihood of classifying patients as clinically distressed increased by 53%. This finding is consistent with studies from psychiatry and palliative care demonstrating that healthcare professionals’ own burden is associated with higher assessments of patient distress (Braun et al. 2022; Engler-Gross et al. 2019). These results highlight the role of emotional strain in psycho-oncology and underscore the need to consider the psychological well-being of psycho-oncology professionals within clinical settings.

With regard to perceived support needs, this study demonstrates that even after an initial consultation, a substantial proportion of patients do not require further care. When considering all hospitalized cancer patients, recent studies indicate that despite high levels of distress, only 15–30% of patients are actually referred to initial psycho-oncological care (Goerling et al. 2024; Weis et al. 2018; Zingler et al. 2025). Our data complement these findings by showing that following an initial psycho-oncological consultation, further support was recommended for 75% of referred patients. Concrete follow-up sessions were scheduled with 50% of these patients. Taken together, this suggests that overall, 10–20% of hospitalized cancer patients exhibited a clinically indicated need for further psycho-oncological support.

These findings imply the central role of the initial psycho-oncological consultation as a diagnostic and selective component within care delivery. Compared with studies that assess patients’ support needs solely on the basis of distress screenings or subjective request for consultation, our results provide important additional guidance in differentiating between patients’ initial support needs and the necessity for further psycho-oncological care.

## Limitations

Several limitations of the present study should be considered. The generalizability of the findings is limited due to the clinical assessments being conducted by a small (*N* = 9), homogeneous (female) group of experts. It should be emphasized that psycho-oncologists’ clinical assessments do not exclusively represent an objective reflection of patient distress and may be shaped by social and institutional factors. Clinicians’ assessments may be influenced by prior experiences or comparative reference standards, for example when determining the threshold for recommending further psycho-oncological support. In addition, structural conditions such as available resources and care mandates may influence clinical assessments. For instance, awareness of limited resources or predefined institutional structures may lead to more restrictive or selective support recommendations. Furthermore, it cannot be ruled out that clinical assessments are affected by interaction effects between psycho-oncologists and patients.

The agreement between patients’ assessments and assessments by psycho-oncologists is only interpretable to a limited extent due to the small number of available patients’ assessments. Further validation using larger and more balanced samples is therefore required. As only psycho-oncological assessments were collected and evaluations by other healthcare professionals were not considered, an interprofessional comparative perspective is lacking. Consequently, the external and interprofessional validity of the findings is limited. It remains unclear to what extent the identified predictors genuinely reflect patients’ levels of distress or instead capture potential assessment biases on the part of psycho-oncologists.

Due to the cross-sectional study design and the single-center inpatient setting, no conclusions can be drawn regarding changes in distress over the course of treatment or institutional differences. Validation of multi-perspective assessments using longitudinal psychosocial outcomes is necessary.

Additional limitations arise from the timing of data collection during the COVID-19 pandemic. Pandemic-related structural constraints as well as increased levels of distress among both patients and healthcare providers may have influenced the implementation of distress screenings and clinical assessments (see Momenimovahed et al. 2021).

## Implications for research and practice

The results demonstrate that initial psycho-oncological assessments are differentiated, clinically meaningful, and directive for subsequent care. Clinical assessments by psycho-oncologists meaningfully complement patients’ self-assessments and may contribute to the optimization of screening and referral processes. In this context, the initial psycho-oncological consultation proves to be a key diagnostic and selective component of stepped care models.

Demographic and disease-related parameters alone appear insufficient for identifying psychological distress. Instead, patients’ desire for consultation should be systematically assessed, and improved information provision as well as the strengthening of patients’ health literacy should be promoted.

Further research should also more closely examine the working conditions of psycho-oncologists and establish institutional structures that enable reflective engagement with clinicians’ own psychological strain.

In conclusion, future studies should integrate assessments from different professional groups as well as from patients themselves. Findings should be validated longitudinally and include both inpatient and outpatient care settings. The overarching aim is to examine the extent to which assessments conducted during the initial consultation predict long-term psychosocial trajectories.

## Data Availability

The data is available on request from the authors.
